# Bipotent mammary stem cells: now in amazing 3D

**DOI:** 10.1186/s13058-014-0480-0

**Published:** 2014-11-27

**Authors:** Renée van Amerongen

**Affiliations:** Section of Molecular Cytology, Swammerdam Institute for Life Sciences, University of Amsterdam, Science Park 904, 1098 XH Amsterdam, the Netherlands

## Abstract

For many decades, developmental biologists and cancer researchers alike have been trying to understand the relationship between the basal and luminal cell compartments in the mouse mammary epithelium. Delineating the mammary stem and progenitor cell hierarchy will provide fundamental knowledge of how cell proliferation and differentiation are orchestrated to build, maintain and regenerate a complex mammalian tissue. Moreover, it is expected to offer insight into the cells of origin for human breast cancer. A new lineage-tracing study has fuelled the discussion as to the existence of bipotent stem cells in the basal layer of the mouse mammary epithelium.

## Background

Despite its simple appearance, the bilayered mammary epithelium contains multiple cell populations that display complex and dynamic behaviors during different stages of development. The large proliferative and regenerative potential stored in the mouse mammary epithelium is assigned to one or more populations of stem and progenitor cells. These can be prospectively isolated by fluorescence-activated cell sorting from the basal and luminal cell fractions, respectively [1-3]. They are consequently distinguished by functional assays [4,5]: while limiting numbers of stem cells have the capacity to generate a complete ductal network upon transplantation into the cleared fat pad, progenitors show the propensity to form colonies in primary *in vitro* cultures.

A long-lived, bipotent stem cell is thought to lie at the base of the mammary epithelial cell hierarchy [6]. However, this model has been challenged by *in situ* lineage-tracing analyses, one of which demonstrated that transplantation can unlock a multi-lineage potential that is not used during normal development [7] and another that questioned the existence of bipotent stem cells altogether by showing that the mouse mammary epithelium is built and maintained by distinct basal and luminal stem cell compartments [8]. A recent lineage-tracing study by Rios and colleagues [9] adds new fuel to the fire by demonstrating the existence of bipotent stem cells in the basal layer of the adult mammary epithelium. And so the discussion is reignited: are mammary stem cells bipotent or not? And what is the reason for this controversy in the first place?

## Multicolor lineage tracing

Rios and colleagues use state-of-the-art lineage tracing technology to track the developmental fate of a luminal (*Elf5*^*+*^) and a basal (*K5*^*+*^) cell population. For this purpose, they develop two novel mouse lines (*Elf5-rtTA-IRES-GFP* and *K5-rtTA-IRES-GFP*), which they combine with a *tetO-Cre* and a *Rosa26*^*Confetti*^ reporter allele [10]. The resulting ternary system allows them to induce stochastic recombination of the multicolor reporter by administering a single dose of doxycycline, resulting in the stable expression of cyan fluorescent protein (CFP), green fluorescent protein (GFP), yellow fluorescent protein (YFP) or red fluorescent protein (RFP). By combining their multicolor tracing approach with a high-resolution, wholemount three-dimensional imaging protocol, the authors can beautifully visualize (and discriminate) adjacent myoepithelial (that is, basal) and luminal cells.

Luminal *Elf5*^*+*^ cells could be labeled in both the pubertal and the adult epithelium, where they contributed to the expansion and maintenance of the ductal epithelial network. However, labeled clones did not expand in size and were lost after a 12-week trace in adulthood, indicating that *Elf5* marks a luminal progenitor cell population. In contrast, *K5*^*+*^ cells in the basal layer of the epithelium gave rise to clonal patches comprising basal as well as luminal cells. Both in the pubertal and in the adult gland, these clones were long lived and showed expansive growth, giving rise to unicolored patches of ductal epithelium.

## Conceptual advances and missed opportunities

Compared to other reporter alleles in which recombined cells are marked by a single color, the multicolor labeling strategy used by Rios and colleagues greatly enhances the confidence with which two adjacent cells can be scored as being clonally related. However, even with a multicolor reporter it is important to achieve low levels of recombination, particularly when it comes down to quantitative analyses (that is, clone size or the number of myoepithelial versus luminal cells belonging to a single clone). In fact, one could argue that the substantial labeling achieved with a single pulse of Cre activity (up to 30% of the epithelium is labeled after 2 days) somewhat obscures the unequivocal interpretation of the experimental data. In this respect, future studies would benefit from performing ‘re-tracing’ [11] to demonstrate the *de novo* appearance of a mixed-lineage clone from *K5*^*+*^ cells within an already existing clone.

Importantly, however, the detection of tethered myoepithelial and luminal cells within the same (that is, unicolored) clone further strengthens the argument that they are derived from a common precursor. In this respect, it is a pity that the authors do not fully exploit their wholemount three-dimensional imaging protocol by showing movies of three-dimensional reconstructed mixed myoepithelial/luminal cell clones. They also fail to take full advantage of the presence of an IRES-GFP marker in their transgenic driver lines. GFP detection would have been especially informative in mixed clones as further support for asymmetric cell division. GFP expression analysis in short, 2-day trace experiments would also have been useful to demonstrate that the initial recombination event indeed occurred in only the basal or luminal cell fraction of interest, as one inherent limitation of the lineage tracing technology using fluorescent reporter alleles is that the earliest signs of recombination in the mammary gland can often only be detected 48 hours after tamoxifen or doxycycline administration.

## Future challenges

Although the findings of Rios and colleagues confirm the previously reported existence of bipotent adult stem cells in the basal layer [7], the question is how these tracing data, particularly those initiated from *K5*^*+*^ cells, should be viewed in light of the previous study by Van Keymeulen and colleagues [8], which clearly demonstrated lineage restriction for *K5*^*+*^ and *K14*^*+*^ cells. One explanation could be the use of extremely high doses of tamoxifen (15 mg administered during puberty) by Van Keymeulen and colleagues. According to Rios and colleagues, far lower doses of tamoxifen already inhibit outgrowth of the ductal epithelium during puberty [9], which is something that we have observed as well. It is conceivable that the bipotent stem cells are affected by high concentrations of tamoxifen, causing them to die (as previously observed for the +4 cells in the intestinal crypt [12]) or to restrict their lineage potential. In this respect, using the *rtTA*/*tetO-Cre*/doxycycline system would be preferential to using a *Cre*^*ERT2*^/tamoxifen-mediated approach. However, this will generally not be compatible with many existing mouse strains and also requires more complex and expensive breeding strategies. On the bright side, using lower concentrations of tamoxifen appears to have only minor, transient effects on the mammary epithelium [13] and, as demonstrated previously for *Axin2*^*CreERT2*^ [7] as well as for *K5*^*CreERT2*^ and *K14*^*CreERT2*^ by Rios and colleagues, this system in itself is compatible with tracing bipotent stem cells.

A second explanation lies in the use of different mouse strains. Despite their often deceptively similar names, their identity and origin can be very different. For instance, between the two of them, Rios and colleagues and Van Keymeulen and colleagues use three different strains to mark the *K5*^*+*^ basal cell population. One is a *Cre*^*ERT2*^ knock-in in the 3′ UTR of the endogenous *K5* locus generated by the Blanpain lab [8], the second is a *K5*^*CreERT2*^ transgenic line containing the human *K5* promoter generated by the Hogan lab [14] and the third is the *K5-rtTA-IRES-GFP* transgenic line developed by Rios and colleagues, which contains a bovine *K5* promoter [9]. The same holds for the *K14-rtTA* and *K14-Cre*^*ERT2*^ lines used in these studies, with origins in the Fuchs [8] and Chambon labs [9]. Although all of these lines mark basal cell populations, subtle (or not so subtle) differences in their expression levels or activity pattern are to be expected. The only way forward is for investigators to be aware of these differences and to properly document and report them.

Finally, unlike the intestine, which is composed of distinct crypt/villus structures in which cells move along a fixed trajectory, the mammary epithelium lacks a stable architectural unit. This complicates both the analysis and interpretation of *in situ* lineage-tracing experiments. As we grapple to gain experimental control over this technology, it becomes apparent that we still need to gain a far better understanding of many of the basic properties of the tissue, including the steady state turnover of the basal and luminal compartments, the differences between growth and maintenance of the pubertal and the adult epithelium as well as between the ductal and lobuloalveolar portions of the gland and the cellular complexity of both the basal and the luminal layer. It will probably take a lot more tracing before we can connect the dots.

## Abbreviations

CFP: Cyan fluorescent protein
GFP: Green fluorescent protein
RFP: Red fluorescent protein
UTR: Untranslated region
YFP: Yellow fluorescent protein
